# Functional Performance Tests, On-Ice Testing and Game Performance in Elite Junior Ice Hockey Players

**DOI:** 10.2478/hukin-2022-000076

**Published:** 2022-09-08

**Authors:** André-Philippe Daigle, Steve Bélanger, Jean-François Brunelle, Jean Lemoyne

**Affiliations:** 1 Department of Human Kinetics, Université du Québec à Trois-Rivières, UQTR, Trois-Rivières, Canada; 2 UQTR Ice Hockey Research Laboratory, Université du Québec à Trois-Rivières, UQTR, Trois-Rivières, Canada; 3 Athletic therapist, Quebec Remparts, Quebec Major Junior Hockey League, Trois-Rivières, Canada; 4 Head strength & conditioning coach, Centre of Physical Activity, Université du Québec à Trois-Rivières, Trois-Rivières, Canada

**Keywords:** fitness assessment, elite ice hockey, hockey analytics, game performance

## Abstract

This study aimed to explore relationships between fitness, on-ice physical abilities and game performance among elite junior male ice hockey players. Twenty-one major junior ice hockey players (18.9 ± 1.4 years old) participated in the study. Measures including five fitness tests (anthropometric measures, pull up test, bench press test, broad jump, vertical jump) and three on-ice skating tests (multi-stage aerobic skating test, 44-m sprint test, and backward skating test) were assessed during their pre-season training camp. Game performance metrics (collected during the regular season) were collected using InStat software. Results of the (on-ice and off-ice) functional performance test protocol and on-ice tests were analyzed by evaluating correlation coefficients in multiple areas of game performance: 1) physical implication (body checks), 2) offensive contribution (expected goals for, types of zone entries), and 3) defensive actions (blocked shots, expected goals against). They revealed that performance in the broad jump test was associated with skating speed. Some significant correlations were also observed between on-ice test performance indicators such as received body checks, expected goals and blocked shots. In summary, results indicate that on-ice test protocols were associated with players’ performance in multiple aspects of the game. Partial correlation analyses revealed that some of these relationships were specific to the player's position. Forward skating was associated with forwards’ offensive play, and backward skating was specifically related with defensemen’s performance (offense and defense). The addition of on-ice physical tests appears essential for interpreting the results of ice hockey players' physical tests and integrating these results into players’ physical preparation and the in-season follow-up.

## Introduction

Ice hockey comprises high-intensity actions that require players to perform at technical and tactical levels. Such demands become a challenge for coaches and strength and conditioning trainers, who must ensure that their athletes stay at the top of their game while respecting their ability to adapt to the demands of a season (Allard, 2021). Rigorous annual planning and sustained physical preparation are therefore major assets in development and performance at the elite level. Over the past fifty years, ice hockey has evolved and the interest among scholars is growing. The seminal work of Cox et al. (1995) paved the way to our understanding of key variables that are required to excel in ice hockey. In this regard, the determinants of optimal functional performance refer to attributes such as aerobic-anaerobic systems, muscular strength-power, speed and agility (Novák et al., 2019). Several studies relate physical abilities to the multiple tasks of ice hockey players. A considerable number of studies have focused on players’ fitness to establish norms (e.g., Cox, 1995), analyze its physiological demands (Chiarlitti et al., 2018; Douglas et al., 2019; Gilenstam et al., 2011) and predict on-ice skating performance (Kokinda et al., 2012; Lignell et al., 2018). Roczniok et al. (2012, 2016) agree with this finding by identifying key physical attributes that are needed in order to excel. A review by Chiarlitti et al. (2021) shows the scholars’ growing interest towards the evolution of such variables in competition and that physical attributes tend to fluctuate according to the rigors of a hockey season, which appeals for the importance of attaining (and maintaining) high fitness standards in order to excel.

Some authors also paid attention to variables such as the workload and volume over a season. Results from their work showed some fluctuations during a season (Allard, 2021; Brocherie et al., 2018; Stanula and Roczniok, 2014). Modern technology such as LPS-GPS systems brought the opportunity to assess players’ attributes and performance with high efficiency. For example, Douglas and colleagues (2019) showed that forwards displayed higher absolute intensity efforts compared to defensemen. Moreover, the workload tended to decrease during a game regardless of the players’ position. Other authors put some attention regarding game workload and performance. Neeld et al. (2021) also showed among NCAA players that the workload assessed in matches was associated with team performance. Despite such recent advances in this domain, the ability of functional performance tests to predict performance during matches still calls for further investigation. As suggested by Nightingale (2013), associations between physical variables and on-ice performance remain inconclusive and need further work in this area. As Nightingale (2013) stipulates, a gap still exists when trying to establish associations between players’ functional performance and their performances in a game. In the same vein, Kniffin (2017) conducted a 14-year retrospective analysis which showed significant associations between physical fitness indicators and variables such as playing time and offensive contribution at the National Collegiate Athletic Association level (NCAA). Certain discrepancies appear to vary, however, depending on the performance indicators under study. Bringing more advanced metrics by using modern technology can help to shed some light on such associations. For example, Modric and colleagues (2019) reported significant correlations between gameplay performance and soccer players ability to sprint in the attesting context. Inversely, in an ice-hockey specific study, Williams and Grau (2020) examined these relationships and reported that off-ice indicators were not strongly associated with players’ point-shares (e.g., a player’s contribution). This suggests that off-ice tests alone might not be sufficient to explain players' performance in a match. Indeed, it is important to identify attributes that are closely linked to a corresponding dimension observed in match situations. In this regard, Huard Pelletier et al. (2021) showed that the relationships between functional performance and game were inconclusive and the choice of indicators combined with modern technology for in-game data collection might be a way to fill this research gap.

### Study Objectives

We believed that results from fitness testing protocols can explain game performance in elite ice hockey. In this regard, this study had three objectives: 1) to analyze relationships between off-ice functional performance fitness tests and on-ice tests, 2) to verify whether players’ fitness measured in off-ice and on-ice was associated with their game performance success, and 3) to verify whether the relationships differed based on the players’ position. An improved understanding of these associations will help strength and conditioning coaches prepare athletes more efficiently and work in collaboration with coaching staffs.

## Methods

### Participants and Procedures

The study sample consisted of twenty-one elite junior players that evolved in the Quebec major Junior Hockey League (QJMHL), one of the three major junior hockey leagues that make up the Canadian Hockey League (CHL), which is among Canada’s best junior leagues. Players came from one team, since our particular aim was to assess their performance in a regular season. Players who took part in the study (100% male, 13 forwards, 8 defensemen) were between 17 and 21 years of age (18.9 ± 1.4 years). Goaltenders were excluded from the study owing to the small sample size and their task-specific characteristics. Players who agreed to participate in the study signed an informed consent form, approved by the researchers’ institution ethics board (CER-20-271-07.05). Participants completed a battery of functional performance tests at a training camp prior to the 2019-2020 season. Testing sessions were conducted on two consecutive days: 1) Day 1: anthropometric measures and functional performance off-ice, and 2) Day 2: on-ice skating tests.

### Measures

#### Functional Performance Tests (Off-Ice)

*Upper body strength*. We measured upper body strength-endurance using two tests: 1) the bench press test, 2) the pull-up test. Each participant had to perform the maximal number of repetitions on a bench press, using an Olympic barbell (20 kg) plus 75% of their body weight. The bench press test protocol^1^ was validated beforehand (Chiarlitti et al., 2018). Each participant had to maintain a cadence of 25 beats/minutes dictated by a metronome. The number of repetitions (n) completed before the athlete could no longer keep up with the cadence was retained for further analysis.

*Pull-Up Test* (The National Hockey League Combine: https://www.sportsnet.ca/hockey/nhl/2019-nhl-combine-results-top-10-drill/). Participants were required to hold the overhead bar with a pronated grip, hands approximately shoulder-width and arms extended. Participants rose by bending their arms until the chin was above the top of the bar, then lowered themselves to the starting position. Only the number of repetitions performed with the correct technique was counted.

*Lower Body Muscular Power*. We assessed muscular leg power using two tests: 1) the broad jump *(*The National Hockey League Combine: https://www.sportsnet.ca/hockey/nhl/2019-nhl-combine-results-top-10-drill/) and 2) the vertical jump (Bracko and George, 2001). For the broad jump, participants stood behind a marked line on the floor with feet slightly apart. It was required to jump as far as possible by landing on both feet without falling. The best score out of three attempts was recorded for further analysis. For the vertical jump, we used the NHL Combine protocol. The participant stood near a wall with a tape measure attached to it. With the feet planted firmly on the ground, the participant had to reach the highest possible mark on the tape with the hand and fingers extended. He then had to move slightly away from the wall so as to bend the arms and knees for a vertical jump. The athlete jumped as high as possible, touching the tape at the maximum height reached.

#### On-ice Skating Tests

*Sprint Tests (Forward and Backward)*. Sprint tests in skating were inspired from the International Ice Hockey Federation (IIHF) Skills Challenge Manual tests (http://webarchive.iihf.com/fileadmin/user_upload/PDF/100Year/IIHF_Skills_Challenge_Manual.pdf). A modified version was used for assessing speed in a forward and a backward sprint test over a distance of 30.48 m, with the acceleration time recorded at 0 to 7.65 m. The participant started on the ice at the goal line and was required to cover the distance as fast as possible. Time splits were measured and recorded with the Brower timing system, with photocells fixed at a height of 1 m.

*Aerobic Capacity*. The aerobic capacity was measured with the Multi-Stage Aerobic Test for Skating (SMAT) (Allisse et al., 2017, 2019). In the SMAT, athletes had to skate back and forth in 60-s intervals between two predefined lines on the ice at a distance of 45 m, with a 30-s recovery period between each 60-s interval and with increasing speed at each 60-s level. Players were required to synchronize with the predetermined tempo until unable to adapt to the increasing speed. Then, VO_2max_ was estimated based on the last completed stage.

### Game Performance Indicators

Players’ game performance was measured by choosing indicators that were collected with the InStat analysis tool (https://instatsport.com/hockey/instat_scout) which had performance markers computed from the InStat video database. Data covered the full 2019-2020 season, in which the Quebec major Hockey League (QJMHL) played 64 regular season games (specific information about players’ playing time is available in Table 2). The time span between fitness tests and the full season was about five months (September to mid-March). As shown in Table 1, we chose game performance indicators that corresponded with players’ speed, strength, power and endurance, resulting in three categories of indicators: 1) physical implication, 2) offensive contribution, and 3) defensive contribution (Table 1). For physical implication, we assumed that the amount of given and received body checks (hits) would refer to players’ physical implication. We measured offensive contribution by considering expected goals which is a catch-all statistic calculated from an algorithm that takes into account shot provenance and other variables in the InStat platform (http://webarchive.iihf.com/fileadmin/user_upload/PDF/100Year/IIHF_Skills_Challenge_Manual.pdf). Possession metrics such as controlled entries (e.g., entering the offensive zone with puck possession) and controlled zone exits (e.g., exiting the defensive zone with the puck) were also considered because of their association with the team’s offensive performance. Defensive contribution was assessed using two indicators, i.e., blocked shots and defensive expected goals, which is the opponent’s expected goals statistic when a specific player is on the ice.

**Table 1:** Performance indicators and their relationships with performance

Performance indicators	Definition	Associations with performance
Physical implication		

Hits against (Ha)	Situations in which a player is hit by an opponent.	Physical implication, risk of injuries
Hits (H)	Situations in which a player hits an opponent (shoulder check).	Physical implication
Offensive contribution		

Expected goals with a player on (xG-on)	Probability of scoring a goal on shot attempts when the player is on the ice	Offensive talent; creating offensive situations under pressure
Controlled entries (CZE)	When a player enters the offensive zone in control of the puck, by stickhandling and passing	Associated with offensive production and team success
Controlled exits (CEX)	When a player exits the defensive zone in control of the puck	Associated with offensive production and team success
Defensive contribution		

Defensive expected goals (D- xG)	Probability of seeing an opponent score when the player is playing in the defensive zone	Defensive efficiency
Blocked shots (BS)	Defensive situations in which the player blocks the opponent’s shots	Defensive contribution

**Table 2:** Descriptive statistics: time of play, off-ice and on-ice tests.

	Total (n = 21)	Defensemen (n = 8)	Forwards (n = 13)
Age (years)	18.9 ± 1.4	19.1 ± 1.5	18.6 ± 1.2
Body height (cm)	180.0 ± 12.0	180.0 ± 5.0	170.0 ± 17.0
Weight (kg)	80.1 ± 5.3	80.3 ± 5.6	79.6 ± 5.2
Games played (n)	45.8 ± 11.6	45.6 ± 13.2	46.0 ± 9.5
Time on ice (mm:ss)	12:44 ± 5.47	14:30 ± 5.57	11:42 ± 5.42
Shifts per game (n)	19.7 ± 4.3	21.6 ± 3.8	18.6 ± 4.4
Bench press test (reps)	18.8 ± 6.2	19.8 ± 5.9	18.1 ± 6.5
Pull up test (reps)	15.1 ± 3.4	15.9 ± 3.4	14.5 ± 3.5
Inverted row (reps)	17.7 ± 6.1	18.9 ± 5.0	17.0 ± 6.8
Broad jump (m)	254.9 ± 12.3	260.0 ± 6.4	251.1 ± 14.6
Vertical jump (cm)	24.5 ± 3.3	24.5 ± 2.5	24.5 ± 3.8
Forward sprint 7.65 m (s)	1.6 ± 0.1	1.6 ± 0.1	1.6 ± 0.1
Forward sprint 30.48 m (s)	4.4 ± 0.1	4.4 ± 0.1	4.4 ± 0.1
Backward sprint 7.65 m (s)	2.4 ± 0.1	2.3 ± 0.1*	2.4 ± 0.1
Backward sprint 30.48 m (s)	6.0 ± 0.3	5.8 ± 0.2*	6.1 ± 0.3
VO^2max^ (ml/kg*min^-1^)	68.1 ± 2.1		68.9 ± 1.9*
		66.8 ± 1.7	

*Significant difference between defensemen and forwards.

### Statistical Analyses

Results for off-ice and on-ice fitness were analyzed using descriptive statistics. Means and standard deviations of all variables were calculated and assumptions of normality of the distributions were tested using the Shapiro-Wilk (SW) test. No violations of normality were observed (SW statistics; *p* > .05). For objectives 1 and 2, we analyzed correlations between off-ice and on-ice tests (O1) and those involving on-ice fitness and game performance (O2). For objective 3, we controlled for the effect for the players’ position (forward versus defenseman) by analyzing partial correlation coefficients on correlations that were previously obtained. Analyses were performed using SPSS software (version 27), with the level of significance set at *p* < .05.

## Results

### Descriptive Statistics

Descriptive characteristics are presented in Table 2. Although some differences suggest a higher volume of play for defensemen, none of these were significant. The number of games played plateaued around a total of 45 games during the season, which was shortened because of the Covid-19 restrictions imposed in 2020. Fitness results were similar when comparing defensemen and forwards, with no significant differences (*p* > .05). For on-ice tests, we observed that defensemen were better in both backward skating tests (*t*_100_ = 2.263, *p* = .039; *t*_25_ = 2.423, *p* = .029) and forwards had higher VO_2max_ values (*t*_vo2_ = 2.394, *p* = .029).

### Objective 1. Relationships between off-ice and on-ice tests

**Figure 1:**
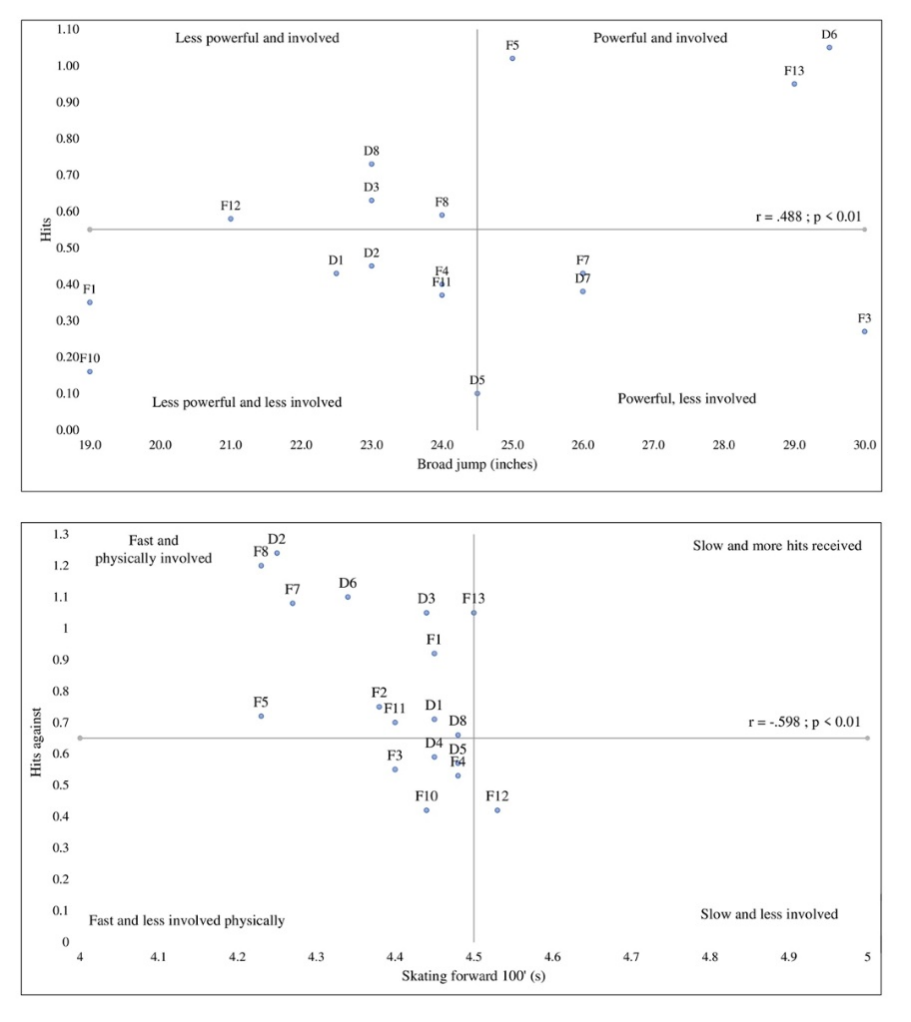
Associations between fitness tests and physical implication (Upper side: Vertical jump and given hits; Lower side: Forward skating speed and received hits).

Correlation matrices are available in Appendix A and B. Two off-ice variables tended to be associated with on-ice physical tests. The broad jump was correlated with the forward skating test (*r* = -.536; *p* < 0.05) and the backward skating test (*r* = -.534; *p* < 0.05). As illustrated in Figure 1, these results show that the most powerful players on the broad jump tended to be faster skaters (*r*_partial_ = -.717, *p* = .003). Furthermore, weight correlated with both backward skating tests (*r*_25'_ = -.617, *p* < .05; *r*_100'_ = -.451; *p* < .05). Partial correlation analyses revealed that this association was significant for forwards and defensemen (*r*_partial_= -.680, *p* < .01).

### Objective 2. Relationships between (off- and on-ice) fitness tests and game performance

We observed only one significant association between off-ice tests and game performance. The only significant association between functional performance and match performance was the association between the vertical jump, and the number of given body checks (*r* = .488, *p* < .05). Partial correlation showed that these associations remained significant even after controlling for the players’ position (*r*_partial_= .512, *p* < .05).

Figure 1 displays correlation coefficients involving on-ice tests and game performance indicators that were found to be significant, while the other correlations were not significant. Figure 1A shows that the fastest players in the forward speed skating test tended to receive more body checks (*r* = -.598, *p* < .05). Inversely, they tended to give more body checks to opponents, yet this association was not significant (*r* = -.209, *p* < .10). When we controlled for the position, partial correlations remained significant (*r_partial_* = -.539; *p* < .05), which means that faster players were involved in play and received more hits, regardless of whether they were forwards or defensemen. In Figure 1B, the negative significant coefficient indicates that the fastest players in the forward sprint test displayed more offensive production with a higher expected goal (*r* = -.485, *p* < .05). However, the non-significant partial correlation coefficient (*r_partial_* = -.397, *p* > .05) shows that this association was specific to forwards.

As with forward skating, Figure 3A shows that greater speed in the backward skating test was significantly associated with offensive production (*r* = -.528, *p* < .05). This association remained significant when controlling for the players’ position (*r_partial_* = -.442, *p* = .05), suggesting that faster skaters tended to contribute offensively for either defensemen or forwards. The results presented in Figure 3B reveal that players who performed better in the backward acceleration test (25 feet) displayed more blocked shots (*r* = -.497, *p* < .05). When we analyzed partial correlations to control for the players’ position, the coefficient was non-significant (*r_partial_* = -.145, *p* > .10) suggesting that such association was specific to defensemen only.

**Figure 2:**
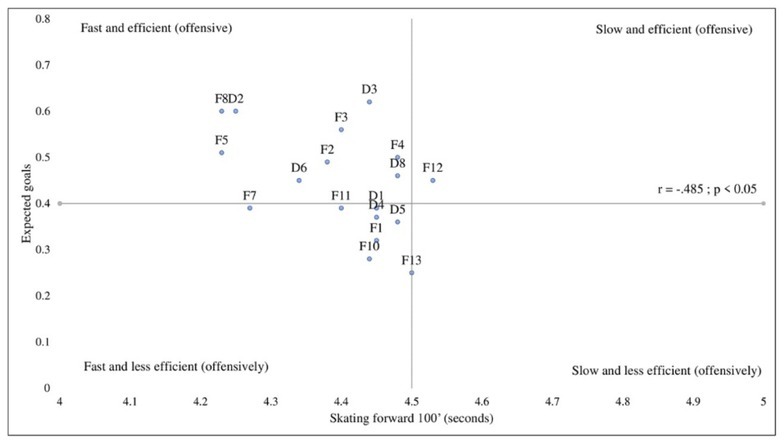
Forward skating speed and offensive contribution (expected goals).

**Figure 3:**
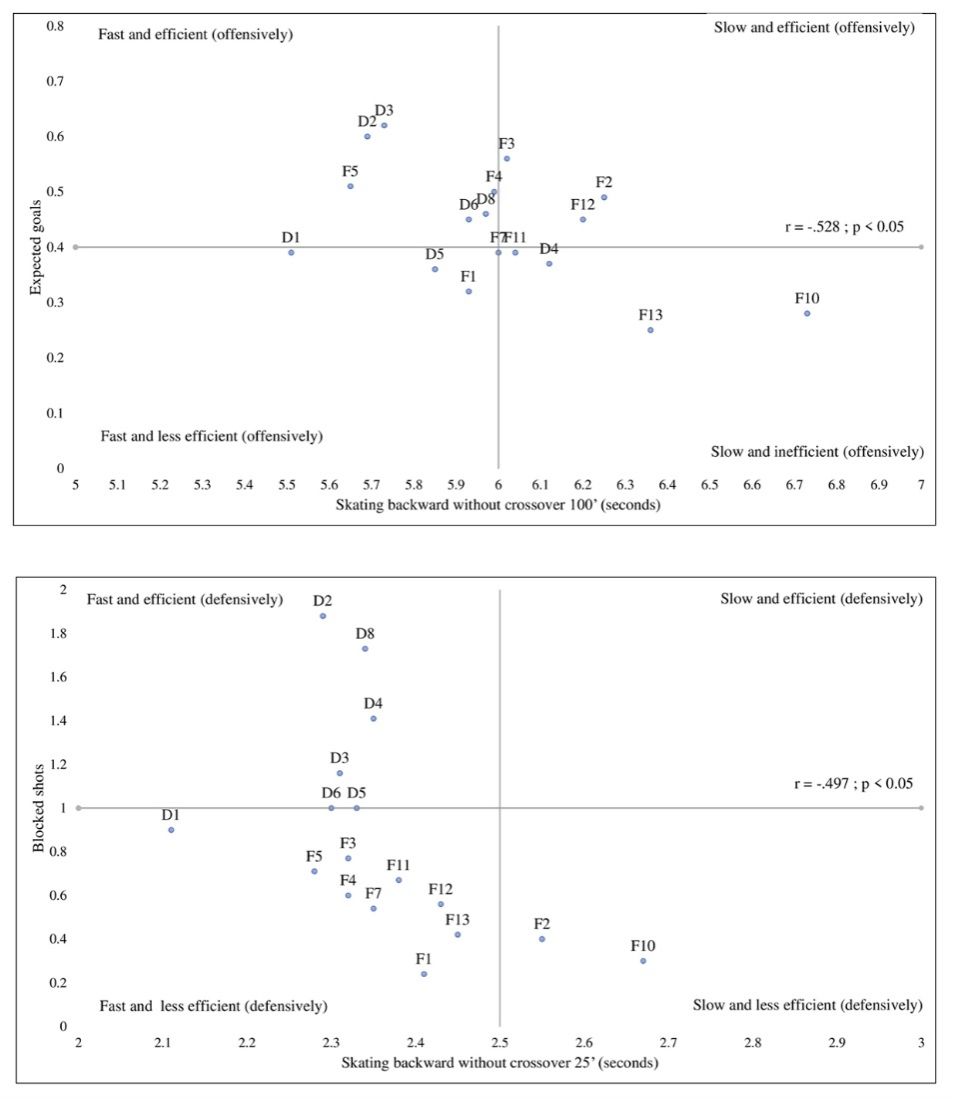
Associations between backward skating speed and game performance (Upper side: Backward skating and offensive expected goals; Lower side: Backward skating and blocked shots).

## Discussion

Preparing hockey players to perform in competition operates through a precise evaluation of their physical abilities. Therefore, we can hypothesize that the fitter the players are, the better they will perform on the ice. In this regard, a wealth of scientific literature documents ice hockey players’ abilities in terms of functional performance and the on-ice performance profile. Despite these contributions, very few authors have studied the relationships between players’ test results and their performance in real game situations. This study offers a new perspective by analyzing results obtained in functional performance tests and their relationships with game performance metrics collected with modern technology.

### Relationships between Fitness and On-Ice Performance

In line with previous research, our results showed few significant relationships between office fitness and on-ice performance. Two off-ice fitness tests were related to on-ice performance. Results of the broad jump test were significantly correlated with on-ice skating sprint tests, which supports the predictive validity of the broad jump for determining skating speed. Such results are in line with the findings of Chiarlitti (2018) and Farlinger (2007), who demonstrated that the forward skating test was correlated with the long jump test. Note, however, that many authors report that muscular power or explosiveness is positively associated with skating speed (Bracko and George, 2001). Our results also show an apparent association between body mass and on-ice performance. Quinney et al. (2008) reported that in terms of ice hockey performance, a dominant lean body mass ratio was associated with muscular power and skating speed, which positively influenced physical performance. In the same vein, Vescovi (2006) studied the anthropometric profile of NHL drafted players between 2001 and 2003 and highlighted the dominance of lean mass over fat mass in top professional prospects. Similarly, Gilenstam (2011) demonstrated a positive correlation between body weight and skating performance in the on-ice acceleration and maximum speed test. The results of these various studies are in line with our results. In fact, such an association seems realistic and suggests that predominant muscle mass is a predictor of on-ice power (Chiarlitti et al., 2018). Our partial correlation analyses, however, show that defensemen in our sample were heavier than forwards, which somewhat diminishes the strength of the association with players’ body mass (Williams and Grau, 2020) and suggests it could be a position-specific association.

### Is Performance in Testing Associated with Success in Games?

As mentioned earlier, there are only few studies that demonstrate strong associations between players’ fitness and their responses in game performance. Consideration of performance indicators that are closely related with corresponding physical attributes seems a way to develop stronger evidence regarding such associations. Our results are congruent with this hypothesis and indicate some positive associations in which physical attributes measured in different contexts (off-ice, on-ice) tend to be related to game performance. First, we observed that performance in the vertical jump was associated with our measures of physical implication in the game (e.g., giving and receiving body checks), suggesting that more powerful players tend to be more physically involved in the game. Although this relationship appears plausible, no other research reveals such findings. In contrast with the conclusions of Kniffin et al. (2017), our analyses did not report significant associations when considering upper body strength and power. We believe that the absence of a significant association may be explained by the lack of specificity between the fitness measures and corresponding performance indicators that we chose.

Secondly, analyses performed with our sample support the relevance of including on-ice tests. Our results revealed that skating speed appeared to be a factor associated with players’ physical implication in the game. In addition, greater speed also resulted in a higher risk of being hit by opponents. While this looks surprising at first, it seems realistic that slower players are less likely to be checked, since they may be less involved in the game. In contrast, a faster player is more likely to carry the puck and get to the puck first in loose puck situations, making him/her more vulnerable to being checked by the opponent. Our results also show that faster skaters (in the forward sprint) tend to generate a higher offensive contribution measured as expected goals. Expected goals (xGs) reflect offensive production and are calculated from the relative contribution of different factors, which allows for calculating the player’s scoring probabilities. We have to specify that xGs are assessed in different ways depending on multiple factors (e.g., passing data, shot provenance, and different situations). In the present study, we used the InStat model which has its own algorithm for calculating xGs. At first, our findings appear to contradict those of Williams (2019) who did not report associations between American college players’ physical attributes on their overall point-share value. It should be noted, however, that the point-share indicator used by Williams (2019) may have been an insufficient indicator. Likewise of interest are the significant associations between the speed of backward skating and the offensive (e.g., expected goals) and defensive actions (e.g., blocked shots) of defensemen. Even though Douglas (2019) stated that the workload of defenders did not vary according to collective performance (expressed in wins or losses), it seems that defenders who are more efficient in backward skating (e.g., speed, acceleration) are also more efficient in specific offensive and defensive actions. Such associations suggest that backward skating is a valid tool for assessing a defenseman’s potential in key actions such as offensive production and blocking shots. In this sense, it is possible for a player to deliver a good performance without the team winning a game, indicating that players’ physical attributes remain factors to be considered when analyzing their performance. Furthermore, we agree with Douglas (2019) that measuring skills related to the player’s agility could add value to the prediction of defensive contribution. In this regard, the relationship between players’ fitness and their position allows us to observe certain aspects more specifically. Based on our results, certain on-ice fitness variables are significantly related to the players’ position. Partial correlation analyses demonstrate that certain relationships appear to operate with respect to the players' position. Accordingly, some tests could be valid depending on the player’s position, which opens the door to a more specific testing approach. Although such approaches can be considered in Canadian football (Yao et al., 2020), there are no studies that confirm the relevance of position-specific testing protocols in ice hockey.

### Summary of Results, Limitations and Future Perspectives

Despite its contribution, this study has some limitations. The sample size is small and even if a sufficient level of statistical power was attained, generalization of our results is limited. Considering one team only (single cohort) may lead to possible bias. For example, the approach involving testing and physical preparation valued by the team under study can relate to players’ responses in fitness and on-ice testing. Better performing players may, in consequence, be evaluated as “best prospects”, leading to more favorable player’s utilization throughout the season. The use of more teams in a research design of this kind would enable a deeper understanding of the associations between the physical attributes assessed and their relationship to game performance. In the longer term, it seems essential to replicate the present protocol with larger samples and even different levels of play. Indeed, it is certain that the results of this study can serve as a baseline for future research.

The second limitation involves the selected performance indicators which were based on our expertise in ice hockey, the rationale supported in the scientific literature and data availability from InStat technology. In our opinion, additional indicators (e.g., skating speed, number of puck recoveries, heart rate, rate of success in one-on-one puck battles) could have been useful for establishing stronger links between players’ physical attributes and their game performance. These objective indicators could serve as additional relevant variables in future research and enable the holistic analysis of physiological demands with game performance.

The third limitation resides in the on-ice tests selected and their potential association with game-specific indicators. In this regard, even if skating speed is important, assets such as skating agility and ability to repeat sprints are key on-ice components that need to be assessed. Measuring these kinds of assets may plausibly lead to important associations with specific aspects of the game. From this perspective, we think that measuring agility (and its related indicators) could reinforce the associations between testing and game performance. As shown by Bracko and George (2001), agility is an important variable to consider when trying to establish a player’s performance profile. Indeed, we think that considering the assessment of this component should be considered to verify its transfer to real settings.

Finally, we believe that the cross-sectional nature (and the time span between players’ testing and the regular season) of the design has its share of limitations as well. Consideration of the statistics covering a full season without taking into account possible fluctuations in players’ performance throughout the season (e.g., even in a single game) could lead to weaker correlation coefficients. A decrease in physical performance over the course of a full hockey season has been observed among junior and collegiate players in the CHL (Chiarlitti et al., 2021; Durocher et al., 2008). This phenomenon, known as athlete’s deconditioning, was not considered in the present study and offers an interesting avenue for future analyses (e.g., does performance decline throughout a season?). In this sense, future research projects on the subject should measure the potential "fatigue" of players by structuring measurement times that verify whether fluctuations in the performance level (physical and on-ice) occur throughout an ice hockey season.

An improved understanding of the relationship between ice hockey players’ off-ice physical abilities and their performance in game situations allows strength and conditioning coaches to develop better training plans and adapt testing methods to ensure that players receive optimal preparation. This study provides some answers especially for choosing tests to prioritize when aiming for peak performance. From this perspective, the long jump test is of first importance because it is associated with the on-ice skills linked with players' offensive play. The addition of on-ice tests makes it possible to bridge the gap between players’ physical fitness and skills and game performance. This study also offers potential further developments. Knowing that some physical attributes are related with game performance, it becomes clearer for strength and conditioning coaches to prioritize which fitness components to train throughout the in (and off) season. From this perspective, approaches based on the specificity of movement (e.g., skating) such as agility training and multiple changes of direction are promising and seem to provide additional benefits (Novák et al., 2021). In this regard, on-ice conditioning seems to be a promising approach for junior elite players who spend large amounts of time on the ice. Off the ice, other approaches also have their potential to enhance players’ predisposition to perform. Some interesting examples prevail in emerging approaches such as post-activation potentiation (Lagrange et al., 2020) and resistance training with focus on eccentric contraction time (Gepfert et al., 2021), which showed benefits among elite hockey players. However, despite the potential of such methods, the benefits to game specific performance remain to be shown empirically with objective game-performance measures (Huard Pelletier et al., 2021).

In summary, including an on-ice physical test in a battery of physical tests to evaluate fitness of ice hockey players is useful, given this test’s potential transferability to game performance. According to our results, strength and conditioning coaches should include on-ice training in their training plans to increase their players’ response to game-specific tasks.
